# Drug screening for ischemic stroke using larvae and adult zebrafish model: a review

**DOI:** 10.1186/s42826-024-00232-4

**Published:** 2025-01-02

**Authors:** Ni Made Dwi Mara Widyani Nayaka, I Ketut Adnyana, Kusnandar Anggadiredja, Indra Wibowo

**Affiliations:** 1 Department of Pharmacology and Clinical Pharmacy, School of Pharmacy, Institut Teknologi Bandung, Jl. Ganesha 10, 40132 Bandung, Indonesia; 2 Department of Natural Medicine, Faculty of Pharmacy, Universitas Mahasaraswati Denpasar, Jl. Kamboja 11A, 80236 Bali, Indonesia; 3 Physiology, Animal Development, and Biomedical Science Research Group, School of Life Sciences and Technology, Institut Teknologi Bandung, Jl. Ganesha 10, 40132 Bandung, Indonesia

**Keywords:** Animal model, *Danio rerio*, Ischemic stroke, Zebrafish

## Abstract

Ischemic stroke (IS) is the most recorded case of stroke that is caused by decreased blood flow to the brain. Nowadays, therapeutical agents for IS are limited and they have not shown maximum clinical results. Therefore, the exploration of new candidates for IS treatment continues to be done. Zebrafish as one of the animal models has its advantages and currently is being developed to be incorporated into the drug discovery pipeline of IS. This review explores the latest applications of the zebrafish model in screening potential therapeutic agents for IS. Key factors related to the experimental design such as developmental stage and strain, routes of drug administration, induction methods, and experimental parameters are also elaborated. Finally, this review offers future recommendations for the use of zebrafish in the pre-clinical study of IS. This review is beneficial as a reference for establishing drug screening protocols using the zebrafish IS model.

## Background

Stroke is the second leading cause of global mortality and threatens survival because of its long-term disability and high economic burden. In 2019, approximately 11% of deaths globally were caused by stroke, which is expected to continue to increase by 2030 [1, 2]. Among all of the stroke cases, 80% were reported to be ischemic stroke. Ischemic stroke (IS) is a complex pathophysiological condition resulting from cerebral thrombosis or embolism leading to neurological disorders [3]. The risk factors for IS include age, sex, race, ethnicity, genetics, current smoking, alcohol consumption, unhealthy diet, and comorbidities such as diabetes, hypertension, and hyperlipidemia [4].

After IS onset, patients are treated with pharmacological therapy or undergo surgery. Only a few clinically approved drugs are available for IS, including recombinant tissue plasminogen activator (r-tPA) injection, antiplatelets, anticoagulants, and neuroprotectants [5]. Exploring new therapeutic agents or treatment strategies for IS that offer better clinical outcomes is carried out through in vitro, in vivo, and clinical research. The pre-clinical stage plays a pivotal role in successful translational studies, and this is greatly influenced by many factors such as the availability of animal models, ethical factors, and the ability of the models to mimic human disease pathophysiology [6, 7]. However, no existing animal model exactly mimics human conditions to date.

Research related to IS has been conducted according to the Stroke Therapy Academic Industry Roundtable (STAIR) guidelines as written in the Standards Regarding Preclinical Neuroprotective and Restorative Drug Development [8–10]. The STAIR’s recommendation is to include experiments in multiple species, meaning that other animal models should be utilized to complement studies using rodents as the standard model. The development of a zebrafish IS model is likely to be part of a scientific effort to fulfill this recommendation [11]. The zebrafish model has been extensively used in health-related research due to its advantages, including easy husbandry, low cost, genetic similarity to humans, high throughput for drug screening, and the ability to perform live imaging observation [12, 13].

From the genetic point of view, 73% of human genome structures have zebrafish orthologue which ~ 47% of them have a single zebrafish ortholog and the rest have more than one zebrafish ortholog. It is ~ 82% of disease-related genes in zebrafish are similar to humans [14, 15]. Related to IS, some genes of zebrafish are homologous with human genes. For example, the Tgfb1b gene of zebrafish is homologous with the TGF-β1 gene of humans. These genes are correlated in terms that they have similar functions in inhibiting inflammation and ischemic injury. In addition, the Il4r.1 gene and the Pla2g4aa gene of zebrafish are homologous with the human interleukin 4 receptor (IL4R) and PLA2G4A, respectively. IL4R is used as a predictor for severe prognosis of acute IS, while PLA2G4A is a marker which upregulated in IS and is associated with the death of neurons [14, 16, 17]. Moreover, when a stroke occurs in humans, there are increased levels of several biochemical markers, such as glutamate, reactive oxygen species (ROS), and inflammatory markers, possibly leading to cell death in the brain [18–20]. These cellular and molecular changes also occur in zebrafish IS models and confirm the conserved pathophysiology of IS in humans and zebrafish.

In this review, we elaborated on the usage of zebrafish as an animal model for ischemic stroke. The critical considerations in designing drug screening procedures including developmental stages, sex, and strain of zebrafish, drug administration protocols, induction methods, and experimental parameters are well discussed. Additionally, we provide an update on new anti-stroke candidates that have been studied with zebrafish models. We also address future perspectives of this model in experimental IS. This review aims to enhance the effective use and understanding of the zebrafish IS model for drug screening purposes.

## Main text

### Zebrafish vs rodent models

**Fig. 1:**
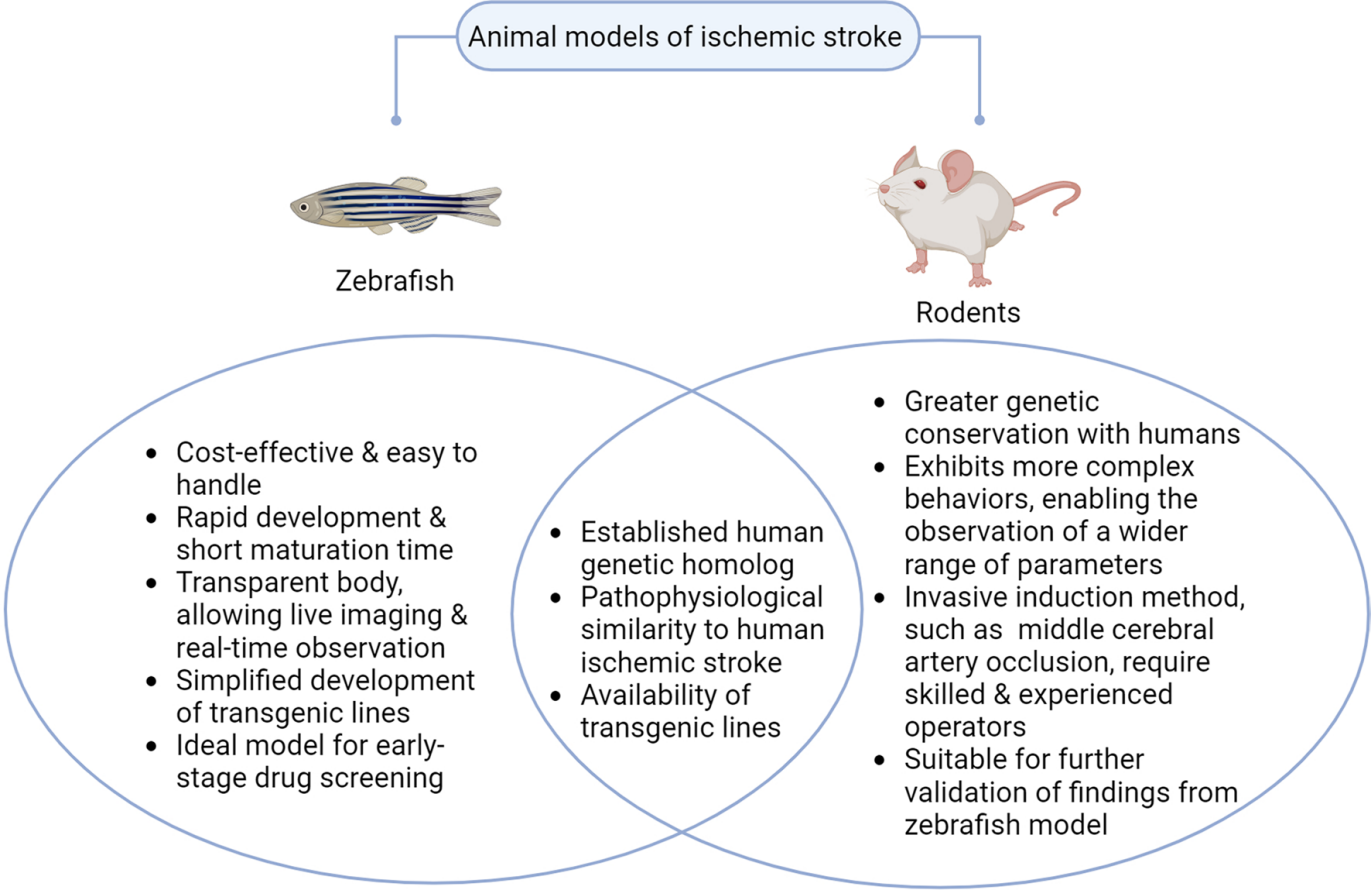
Comparison between zebrafish and rodent ischemic stroke models. Created with BioRender.com

During IS in humans, the levels of key biochemical markers such as glutamate, ROS, and inflammatory cytokines rise significantly, contributing to neuronal cell death and brain damage [18–20]. Similar cellular and molecular alterations are consistently observed in zebrafish and rodent models of IS. Notably, zebrafish share many neuroanatomical and physiological characteristics with mammals, including neuronal morphology, synaptic circuitry, and overall nervous system organization [21]. These similarities form the basis for the extensive use of zebrafish in studying cognitive behaviors and locomotor deficits across a range of neurological disorders, including motor neuron diseases [22, 23]. In humans, motor dysfunction is a common consequence of IS [24]. Zebrafish and rodent models of stroke exhibit analogous cognitive and motor impairments, further supporting their validity in replicating the pathophysiological processes of human IS. Figure 1 presents a comparison between zebrafish and rodent models of IS.

Several IS induction methods established in rodent models have also been adapted for use in zebrafish, including the photothrombotic technique and endothelin-1 induction [25–28]. Additionally, other zebrafish IS models, such as ponatinib-induced and hypoxia-induced methods, offer simplicity and ease of execution (for further details, refer to the section on 'Induction methods'). In contrast, the standard induction procedure in rodent models, particularly middle cerebral artery occlusion (MCAO), requires invasive surgical techniques that demand a high level of skill and operator experience [29].

The zebrafish model offers significant advantages for IS research, including its cost-effectiveness, ease of handling, and high-throughput screening capabilities, rendering it particularly useful in the early stages of drug discovery. Transgenic lines used to study ischemic stroke are available for both rodent and zebrafish models, such as C57Bl/6, Sv/129, C57BI/6, FVB/N, and HuR transgenic mouse lines [30–32] and Tg(JS7:EGFP), Tg(MPO:GFP), and Tg(HuC:Kaede) zebrafish transgenic lines (further discussion in the section 'Zebrafish strains'). However, zebrafish offer the advantage of easier genetic manipulation, enabling the rapid development of transgenic lines specifically tailored to investigate targeted aspects of IS treatment. The transparency of zebrafish embryos and larvae enables live, real-time imaging of biological processes, a capability that is challenging to achieve in rodent models. Nonetheless, zebrafish, particularly in the larval stage, lack the behavioral complexity observed in rodents, thereby limiting their utility in assessing post-stroke cognitive and functional deficits. Moreover, rodents exhibit greater physiological and neuroanatomical similarity to humans, enhancing their relevance for translational studies to predict clinical outcomes and currently being the standard model for in vivo study of IS.

Several studies have demonstrated comparable outcomes between zebrafish and rodent models in preclinical drug screening for IS. For instance, Guhong injection was found to ameliorate brain function in a ponatinib-induced zebrafish model [33], consistent with findings in a MCAO mouse model [34]. Similarly, minocycline, a potential therapeutic agent for IS, was shown to reduce infarct volume and improve motor function in both zebrafish and rodent models [35]. However, while glutamate suppression was observed in the MCAO mouse model, this effect was absent in the hypoxia-insult zebrafish model, highlighting potential differences in pathophysiological responses between species. These findings suggest that while zebrafish models offer valuable insights into early drug discovery, they should not be used in isolation. Confirmatory studies using rodent models are crucial, as both models have inherent advantages and limitations. The complementary use of both models can enhance the translational relevance and robustness of preclinical findings.

### Designing experiment using the zebrafish ischemic stroke model

**Fig. 2:**
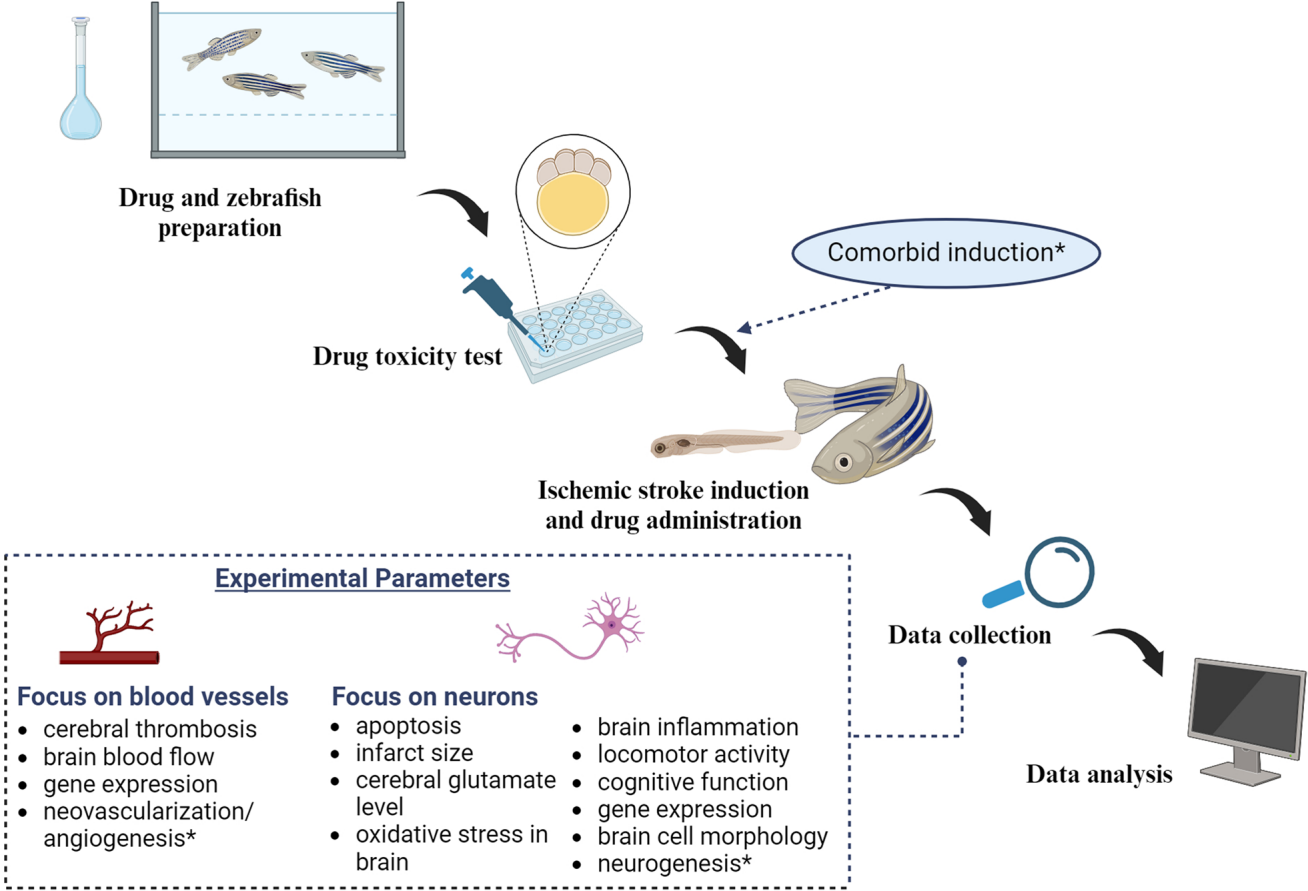
Schematic diagram of the experimental drug screening design using a zebrafish ischemic stroke model. *Future development of this model. Created with BioRender.com

Table 1 demonstrated that the zebrafish models responded to human IS therapeutics such as Activase®, aspirin, edaravone, clopidogrel, Naoxintong capsules, Shuxuening injection, and Xingnaojing injection. Therefore, the models are considered to be reliable and effective to be used for drug screening purposes. Figure 2 summarizes the key steps in designing a drug screening protocol for experimental IS using the zebrafish model including the preparation of zebrafish and drug, acute toxicity test using fish embryo, disease induction, data collection, and data analysis. In this section, we elaborate on the essential considerations for designing experiments with the zebrafish IS model.

**Table 1:** Recent development of zebrafish ischemic stroke model

Type of model	Adult/ Larva	Strain/Type	Induction method	Type of model	Main findings	References
Focal ischemic stroke	Adult	NA	Photothrombotic (i.p Rose Bengal 100 µg/g BW, light intensity of 800 μW/cm^2^, 20–30 min)	Focal	The photothrombotic treatment in zebrafish induced cerebral thrombotic ischemic damage, particularly in the optic tectum, resulting in abnormal locomotor behavior. This model showed a positive response to Activase®, suggesting that the cerebral damage was due to blood vessel occlusion in the brain, effectively mimicking focal cerebral ischemia observed in humans	[27]
Acute ischemia stroke	Adult (5–6 months)	Wild-type	Hypoxia (N_2_ gas, ± 0.6–1.8 mg/L DO, 10–25 min)	Global	Zebrafish can be established as a model for investigating sex-specific neural damage and repair caused by hypoxia induction. Female zebrafish exhibited greater susceptibility to hypoxic conditions but demonstrated a more rapid recovery than males. This model holds the potential for advancing therapeutic strategies for stroke treatment in female patients	[38]
Ischemic stroke	Larvae (2 dpf)	Albino strain (mutant in slc45a2)	Ponatinib (1 µg/mL, 24 h)	Global	The ponatinib-induced zebrafish model closely mimics the human pathophysiology of IS through mechanisms such as cerebral vascular endothelial injury, cerebral thrombosis, reduced cerebral blood flow, neuronal apoptosis, cerebral inflammation, and decreased motility. This model demonstrated responsiveness to human pharmaceuticals, including aspirin (3.125–100 µg/mL), clopidogrel (0.94–15 µg/mL), Naoxintong capsules (3.9–62.5 µg/mL), edaravone (1.56–25 µg/mL), Xingnaojing injection (0.78–10 ng), and Shuxuening injection (2.2–35 ng)	[51]
Mini stroke	Adult (5–8 months)	Wild-type (TL/AB) strain	Preconditioning hypoxia (Intermittent treatment: 0.6–2 mg/L DO, 5–10 min for 3 months; Acute treatment: 0.6 mg/L, 5 mi)	Global	Zebrafish with preconditioning hypoxia were established to mimic mini-strokes in humans and have a neuroprotection effect. Female zebrafish demonstrated a superior capacity for adaptation to preconditioning hypoxia compared to their male counterparts	[40]
Ischemic stroke	Larvae (4 dpf)	Wild-type & Tg (HuC:Kaede) strain	Hypoxia with oxygen absorber (Anaero Pack®, 3.0 mg/L DO, 2 h)	Global	Hypoxia induction using an oxygen absorber simulated ischemic stroke by causing head edema and increased neuronal cell damage. This method offered higher throughput compared to the N_2_ perfusion technique and aligned with mammalian models, as it responded similarly to treatments with edaravone (500–1000 mM) and MK-801. The Tg (HuC:Kaede) strain facilitated the detection of neuronal cell damage in the brain without the need for staining, thereby expediting the experimental process	[39]
Diabetes + Global ischemia	Adult (3–4 months)	Pink type	D-glucose (111 mM) + Endothelin-1 (3 µl/gm)	Global	Diabetic groups experienced altered endothelial function and exhibited greater impairments in locomotion and memory relative to normal groups. These findings indicate that diabetes exacerbates the effects of stroke events. It is relatable to the human condition where diabetes is a known risk factor for IS	[28]

*DO* dissolved oxygen, *dpf* days post fertilization, *i.p* intraperitoneal, *IS* ischemic stroke, *NA* not available

### Induction methods

Inducing ischemia in zebrafish can result in two types of models: focal and global. The global model is characterized by damage not only in the brain but also in other organs, such as the heart, whereas the focal model restricts ischemia to brain tissue [36]. Several techniques are used to replicate IS in zebrafish, including ponatinib-induced, hypoxia-treatment, and photothrombotic-induced methods. Based on our review (Tables 1 and 2), ponatinib and hypoxia treatments are the most frequently used methods due to their procedural simplicity.

**Table 2:** Recent exploration of new therapeutic agent for ischemic stroke using zebrafish model

Sample/Drug candidate	Drug administration	Adult/Larva	Strain/type	Induction method	Mechanism and targets*	References
Magnesium L-threonate (1–10 mM)	p.o, before induction	Adult (4–6 months)	NA	Hypoxia (Gaspak™, 1.0 ± 0.5 mg/L DO)	Cognitive function↑; infarct size↓	[16]
Isoliquiritin (50–100 mg/kg)	i.p, after induction	Adult (7–10 months)	Wildtype AB strain	Photothrombic (i.p Rose Bengal 50 µg/g BW, light intensity ≥ 1600, 000 LX, ≥ 3200 K for 30 min)	Infarct size↓; repair brain cell morphology (H&E staining); locomotor behavior↑; antioxidation: SOD↑, GSH-Px↑, MDA↓; transcriptomic analysis: 25 key DEGs (*angptl3, bcl10, bcl3, bcl2l11, casp6, casp8, cldnh, cxcl8a, fadd, itgb3a, il1b, il6, il10, irf3, ifnphi1, jak1, LOC100537553, mmp9, nfkb1, stat3, tjp3, tnfsf18, tp53, traf6, tradd*)	[36]
Guhong injection (2.5–7.5% v/v)	Bath immersion, co-treatment with induction	Larva (6 dpf)	Wildtype AB strain	Ponatinib (1 µg/mL, 24 h)	locomotor behavior↑; anti-inflammation: NF-κB↓, TNF-α↓, IL-1β↓; coagulation cascade: F7↓, F2↓	[33]
Brazilin (20–50 µM)	Bath immersion, after induction	Larva (6 dpf)	NA	Hypoxia (N_2_ gas, < 0.3 mg/L DO)	Locomotor behavior↑	[91]
Minocycline (1 mg/L)	Bath immersion, after induction	Adult (4 months)	Wild type	Hypoxia (N_2_ gas, 1.5–1.7 mg/L DO)	Infarct size↓; locomotor behavior↑	[35]
Qilong capsule (1.56–25 µg/mL)	Bath immersion, co-treatment with induction	Larva (2 dpf)	Wild-type AB	Ponatinib (1 µg/mL, 24 h)	Area of cerebral thrombosis↓; red blood cell staining intensity↓; coagulation-related genes expression: *PCK*-α↓, *PCK*-β↓, *fga*↓, *fgb*↓, *f2*↓, *vWF*↓; vascular endothelium-related genes expression: VEGFR↑, vegfaa↑	[50]
		Larva (2 dpf)	Wild-type AB	Ponatinib (1 µg/mL, 24 h)	Brain cell apoptosis↓; inflammation-related genes expression: *il-10*↓, *il-1β*↓, *p38*↓, *nf-κb*↓; apoptosis-related genes expression: *caspase1*↓, *caspase2*↓, *caspase3*↓, *caspase7*↓, *caspase8*↓*, caspase*9↓, *bax gene*↓	
		Larva (2 dpf)	JS7 transgenic zebrafish	Ponatinib (1 µg/mL, 24 h)	Macrophage aggregration↓; number of macrophages↓	
95% ethanolic extract from red heartwood part of *Caragana jubata* (2–50 µg/mL)	Bath immersion, co-treatment with induction	Larva (2dpf)	NA	Ponatinib (1 µg/mL, 24 h)	Cerebral thrombosis incidence↓	[65]
6S-5-Methyltetrahydrofolate-Calcium (497.5 mg/kg)	i.p, after induction	Adult (7–10 months)	AB strain	Photothrombic (i.p Rose Bengal 5 µL/0.1 BW, light intensity ≥ 1600, 000 LX, ≥ 3200 K for 20 min)	Telencephalon injury score & rate↓, locomotor behavior↑; antioxidation: SOD↑, GSH-Px↑, MDA↓; transcriptomic analysis: 20 key DEGs (*adamts3*, *adamts8a*, *adamts9*, *capn2a*, *crfb12*, *f3a*, *f3b*, *gadd45aa*, *hspb1*, *il11ra*, *il4r.1*, *il6st*, *lifrb*, *mapk14a*, *met*, *mmp9*, *nfatc1*, *pla2g4aa*, *tgfb1b*, *LOC100334116*)	[17]

*BW* body weight, *CAT* catalase, *DO* dissolved oxygen, *dpf* days post fertilization, *GSH-Px* glutathione peroxidase, *H&E* hematoxylin–eosin, *i.p* intraperitoneal, *MDA* malondialdelhyde, *p.o* per oral, *NA* not available, *ROS* reactive oxygen species, *SOD* superoxide dismutase

^*^↑: activation or upregulation; ↓: inhibition or downregulation

Ponatinib, a tyrosine kinase inhibitor used in cancer treatment, has thrombosis as a major side effect, making it a useful agent for inducing ischemic events in zebrafish models [37]. The cerebral thrombosis induced by ponatinib mimics the pathophysiology of IS in humans, where a thrombus obstructs blood vessels. While this model is relatively easy to establish, it has limited relevance to clinical ischemic stroke due to the global damage observed in zebrafish. However, we propose that this model can still serve as a useful preliminary tool for studying new anti-thrombotic therapies.

In hypoxia treatment, zebrafish are immersed in low-oxygen water which results from nitrogen perfusion or oxygen absorption [38, 39]. Compared to nitrogen gas, the oxygen absorber had better performance in decreasing the dissolved oxygen level and simplifying the induction procedure. Das et al. established a new model which aimed to mimic mini-stroke in humans. In their model, hypoxia was preconditioned for 3 months, followed by severe acute hypoxia treatment [40]. This experiment aimed to develop a zebrafish model mimicking mini-stroke in humans. Conversely, cerebral injury caused by hypoxia can be spontaneously reserved when the fish are exposed under normal conditions. As a member of the *Cyprinidae* family, zebrafish are tolerant to low-oxygen environments and exhibit adaptive abilities [41]. Therefore, designing a precise observation time window is a key factor in obtaining accurate data. It is worth noting that in the 24 h after hypoxia induction, brain damage in zebrafish showed a slight increase [35], which in humans is indicative of reperfusion injury after receiving thrombolytic treatments. Thus, this model can be used to screen new therapeutic agents to reduce brain damage after recanalization.

Photothrombotic treatment, previously performed in rodents, is also applicable in zebrafish models [27]. Rose Bengal, a photosensitive dye, is injected before induction, and it reacts to green excitation light (560 nm) that illuminates the brain directly in zebrafish [36, 42]. This reaction produces ROS and triggers a cascade of coagulation, resulting in ischemic lesions that mimic the clinical pathology of stroke. Compared to other IS induction methods, this technique is argued to be superior in controlling the thrombosis area, whether it is a partial or complete occlusion, producing a focal ischemic stroke at a specific part of the brain [43]. Furthermore, the induction procedure in this method involves the thrombus and vascular embolism formation, which represent clinical situations [44]. However, the photothrombotic method has a similar drawback to the permanent MCAO in rodent models considering their inability to mimic reperfusion conditions.

Further discussions on these induction methods have been reviewed in previous studies [45, 46]. On the other hand, a new induction method generating a more complex zebrafish IS model by Chavda et al. [28] has not been reviewed elsewhere. They successfully established a zebrafish model of IS incorporating diabetes, a condition associated with an increased risk of stroke. In their study, chronic hyperglycemia was induced in zebrafish using D-glucose to simulate type 2 diabetes mellitus, followed by oral administration of preproendothelin-1 (ET-1) to induce IS. This model mirrors the human condition, where elevated ET-1 levels in diabetes may increase the incidence of cerebrovascular stroke through a complex pathophysiological process. Compared to other groups, the diabetic ET-1-treated group exhibited the most severe brain injury and behavioral impairments. These findings suggest that diabetes exacerbates stroke severity, opening new avenues for using a comorbid zebrafish model in experimental IS. Additionally, since each induction method models different aspects of IS in humans, we recommend the usage of combined models to better capture the complex patophysiology of IS. This approach would strengthen the positive outcomes of drug candidates. Moreover, confirmation of efficacy in higher animal models should be considered when using zebrafish models to ensure translational relevance.

### Developmental stages

It is important to consider the developmental stages of zebrafish when designing an IS experiment to ensure method reproducibility. In general, there are four developmental stages of zebrafish: embryo, larvae, juvenile, and adult. Zebrafish embryos hatch in 48–72 h post-fertilization (hpf) which indicates the starting point of the larval period [47]. During the 6 weeks of larval stage, the fish grow three times longer and experience some morphological transformations to become juveniles. The juvenile fish will lose their larval fin fold and develop scales. By around 3 months, sexual maturity will be acquired and the fish finally enter the adult stage. It is important to note that the developmental rate of zebrafish is majorly modified by environmental aspects, for example, temperature, water quality, and fish density [48, 49].

**Fig. 3:**
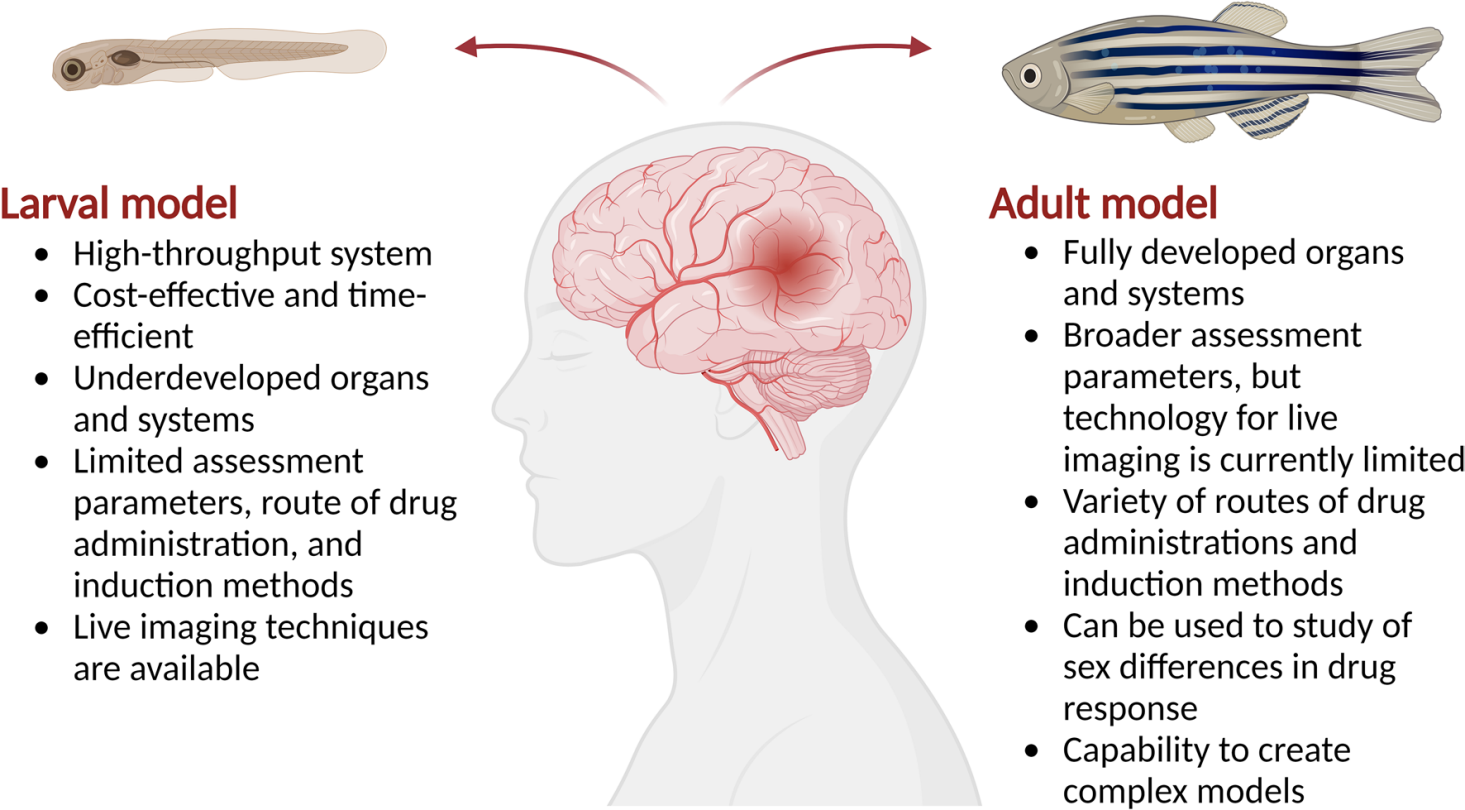
Comparison between larval and adult zebrafish ischemic stroke models. Created with BioRender.com

In experimental IS, postembryonic stages are preferred due to the maturation of the neurovascular system. As shown in Table 1, both larval and young adult stages of the zebrafish IS model have proven reliable, demonstrating responsiveness to clinically approved IS treatments. They also have been used to screen new therapeutic agents for IS (Table 2). Moreover, the key differences between larvae and adult zebrafish and their application in experimental IS are summarized in Fig. 3 and Table 3, respectively. Although adult zebrafish present the advantage of fully developed organs, the larval stage may be more effective for accelerating the drug screening process, as it supports the model's high-throughput capabilities.

**Table 3:** Developmental stages of zebrafish and its usage on drug screening using zebrafish ischemic stroke model

Developmental stages	Development landmarks related to zebrafish IS model		References
*Larva*			
2 dpf	Maturation of brain blood vessels, presence of functional platelets and coagulation factors	Can be used to predict anti-cerebral thrombosis of novel compounds	[50–52]
4 dpf	Maturation of swimming ability	Can be used to observe the drug’s effect on restraining locomotor impairments	[53]
6 dpf	Locomotor activity is more established	More parameters to study locomotor behavior could be observed	
10 dpf*	Formation of BBB	Can be used to predict the BBB permeability of drug candidates	[54]
*Adult*			
≥ 3 months	Organs and systems are fully matured	Applications as shown in Fig. 3	
39 months*		Can be used to mimic human patients regarding age since it is equivalent to approximately 80-year-old humans	[55]

*BBB* blood–brain barrier, *dpf* days post fertilization

^*^Future development of the model

Larvae between 2 and 6 dpf were commonly used in the experiments. The selection of 2 dpf larvae is particularly advantageous because brain blood vessels have matured by this stage, and functional platelets and coagulation factors are present [50–52]. Swimming ability matures at 4–5 dpf, making this the optimal stage for locomotor activity analysis [53]. The blood–brain barrier (BBB) begins to develop around 2.5 dpf and is fully formed by approximately 10 dpf [54]. Therefore, research related to the ability of drug candidates to penetrate BBB in IS model should be done with larvae later than 10 dpf. Furthermore, according to previously established age equivalencies, 7-month-old zebrafish are roughly comparable to 15-year-old humans, while 39-month-old zebrafish approximate the age of 80-year-old humans [55]. Considering stroke usually occurs in older people [56], adult zebrafish (39 months) might have a better representation of human patients. On the other hand, experimental investigations of IS have not utilized zebrafish at either 10 dpf or 39 months of age. This future exploration is possible because it has been used in drug discovery for other diseases [57, 58]. In addition, the use of 39-month-old zebrafish may be challenging because the average lifespan of zebrafish is 36 months, although their lifespan can be extended up to 5 years in the laboratory under controlled conditions [55].

### Zebrafish sex

Sex is among the many nonmodifiable risk factors contributing to IS in humans. Stroke usually occurs more often in women than men [4]. Some experiments have used zebrafish IS models to compare the survival ability of males and females concerning brain damage. After hypoxia, female zebrafish exhibited greater neural damage than their male counterparts [38]. Another study revealed that female zebrafish showed faster neural adaptation than males after being induced with preconditioning hypoxia [40]. A later study from Das et al. [59] confirmed that early neurogenic markers were silenced by the upregulation of H3K9me3 levels in male zebrafish, thus slowing their recovery phase from hypoxia. In humans, H3K9me3 is an important histone-based epigenetic regulatory mechanism that plays a role in regulating the formation of new neurons in adults during the recovery phase after hypoxia–ischemia. These results suggested that the zebrafish model is useful for elucidating the mechanisms underlying sex disparities in IS. This model can also be used to study differences in drug effects on women and men, which have not currently been explored.

### Zebrafish strain

Several strains of zebrafish are available as animal models for experimental IS, including wild-type, albino, and various transgenic strains. Among these, wild-type zebrafish are the most commonly used strain in the reviewed studies (Tables 1 and 2). There are multiple wild-type strains, such as AB, Tübingen long fin, and the wild Indian Karyotype [60]. Given that different zebrafish strains may exhibit variations in experimental outcomes, it is recommended that the strain used in IS modeling be standardized. However, this has not yet been widely adopted in the field.

In addition to wild-type strains, the zebrafish model offers various transgenic strains tailored to specific research objectives [61, 62]. For instance, the JS7 line or Tg(JS7:EGFP) strain is a transgenic zebrafish line that expresses enhanced green fluorescent protein (EGFP) [63]. It has been used to analyze brain inflammation due to its ability to express macrophage-specific fluorescence. A study using larvae from this transgenic line showed that ponatinib-induced IS zebrafish exhibited increased macrophage migration in the brain compared to the control group, indicating cerebral inflammation [50]. The Tg(MPO:GFP) strain is another zebrafish transgenic line employed to study inflammation, as it specifically labels neutrophils. Analysis using this transgenic model revealed an increase in fluorescent neutrophils in the brains of ponatinib-induced IS zebrafish, while in control zebrafish, neutrophils were primarily localized within the blood vessels [51]. This finding suggests the presence of brain inflammation following IS induction.

Cell death in the brain following ischemic insult is typically confirmed by acridine orange (AO) staining [39, 50]. However, AO staining has limitations in distinguishing specific cell types. The Tg(HuC:Kaede) zebrafish transgenic line offers an alternative by using Kaede expression to label neurons [64]. Kaede is a fluorescent protein with photoconversion properties, allowing it to shift from green to red fluorescence upon UV or violet light exposure. This transgenic model enables the identification of dead neurons in the brain without the need for staining. A decrease in Kaede fluorescence intensity indicates greater neuronal damage [39]. Further information on transgenic lines and imaging targets relevant to experimental IS using the zebrafish model has been presented elsewhere [45].

### Route of drug administration

As a tool for preclinical study, drug solutions can be administered to zebrafish before, during, and after stroke induction through different routes, including bath immersion, oral injection, and intraperitoneal injection [16, 51, 65]. Table 2 shows that the bath immersion method is the most popular technique for drug administration in drug screening routines. This technique is effortless, as it simply consists of pouring the drug solution into a water medium, after which the drugs are absorbed by the fish through the skin or ingestion [66]. On the other hand, the bath immersion method does not apply to hydrophobic drugs and is unable to control the exact extent of drug absorption [67, 68]. It is worth noting that the bath immersion method does not represent the actual conditions in clinical practice. Therefore, we suggest using this route of administration only for preliminary studies of drug discoveries. For further examination, we recommend using more advanced techniques.

Oral and intraperitoneal routes are examples of advanced techniques for administering drugs to zebrafish. They only apply to adult zebrafish and can be used to distribute hydrophilic and lipophilic drugs [69]. Before oral administration, the zebrafish were anesthetized using agents such as tricaine solution [70]. The amount of drug solution given orally should be less than 1% of the fish's body weight, usually 1 μL [66]. Kim et al. reported that volumes greater than 2 μL could cause regurgitation [16]. After being given orally, the drug solution enters the gastrointestinal tract and is then distributed through the zebrafish bloodstream. Meanwhile, during intraperitoneal injection, the drug is administered into the abdominal cavity, posterior to the pelvic girdle of zebrafish [71]. Although both methods might have better control over drug absorption, execution needs experience and skill to prevent the risk of injuring the fish’s internal organs.

### Experimental parameters

Based on the studies included in our review, the anti-IS effects of drug candidates have been evaluated using various parameters, including cerebral thrombosis, gene expression, apoptosis, infarct size, oxidative stress and inflammation in the brain, locomotor activity, and cognitive function. Due to the immaturity of their organs and systems, zebrafish larvae may pose limitations for certain assessment parameters, such as infarct size, which can only be accurately measured in adult fish. However, the transparency of zebrafish larvae enables live and real-time imaging. The parameters of the experimental zebrafish IS model have been thoroughly documented in the previous review by Chen et al. [45]. In this section, we focus exclusively on the parameters not covered in their review such as cerebral thrombosis and transcriptomic analysis. Other parameters, such as infarct size, oxidative stress, and inflammation in the brain, can be measured using standardized techniques.

To determine the antithrombosis effect, the experimental zebrafish larvae were stained using the o-dianisidine solution. The dye of o-dianisidine will react with red blood cells producing a dark brown precipitate useful for identifying erythrocytes [50]. Following the staining procedure, the head area was observed under a stereomicroscope, and the images were analyzed using software. The effects of the drug candidates on reducing cerebral thrombosis were quantified as cerebral thrombosis incidence, relative area of cerebral thrombosis, and red blood cell staining intensity [50, 51, 65]. If the wild type of zebrafish is used, depigmentation procedures are recommended as the pigment might interfere with the thrombosis observation.

The transcriptome refers to the whole set of RNA molecules transcribed by certain tissues or cells, including protein-coding (mRNAs) and a variety of noncoding RNAs (ncRNA), such as tRNA, rRNA, lncRNA, and pri-miRNA. Transcriptomic analysis allows the determination of gene functions and structures at the whole level and the elucidation of specific molecular mechanisms involved in diseases [72, 73]. RNA isolated from samples are further analyzed using established methods such as expressed sequence tag method, serial and cap analysis of gene expression, microarrays, and RNA-Seq [74]. In drug discovery, this method enables researchers to comprehend the molecular mechanism of drug candidates, highlighting their potential to impact gene targets. Some studies have isolated the RNA from the brain of the zebrafish model to reveal the effect of isoliquiritin [36] and 6S-5-methyltetrahydrofolate-calcium [17] on regulating genes related to IS (for further details, refer to the respective drug descriptions under the Sect.‘Drugs screened using the zebrafish model of ischemic stroke’).

### Drugs screened using the zebrafish model of ischemic stroke

Pharmacological treatments for IS aim to mitigate acute neuronal damage, thereby reducing the risk of death and long-term disability. These treatments also work to prevent secondary complications arising from patient immobility and neurological dysfunction, as well as to lower the likelihood of future stroke occurrences [5]. In general, there are two primary strategies for treating IS: one targets the vascular system, and the other focuses on neuroprotection. Vascular-targeted therapies, including thrombolytic agents, antiplatelets, and anticoagulants, are primarily employed for recanalization, which is critical for restoring blood flow and ensuring oxygen supply to the brain tissue. In contrast, neuroprotective agents play a crucial role in preserving neurons from the cascade of ischemic damage and safeguarding the penumbra region before reperfusion, thereby preventing the expansion of the infarct area [75]. Using the zebrafish IS model, experiments are designed to evaluate the potential of drug candidates to address both strategies. As the development of this model is still ongoing, only a few new drug candidates have been studied in the zebrafish IS model to date, as shown in Table 2.

### Magnesium L-threonate

Some studies exhibited the effect of magnesium L-threonate (MgT) on neuronal survival [76, 77]. While magnesium may improve cognitive function through the enhancement of synaptic plasticity, threonate plays an important role in inducing magnesium ion transport into hippocampal neurons. Moreover, Slutsky et al. [78] demonstrated that MgT could enhance memory recall. The neuroprotection effect of MgT has been studied in the zebrafish IS model by Kim et al. [16]. In their study, zebrafish pretreated with MgT maintained a preference in time, distance, and frequency of entries to the target compartment after hypoxic insult. The 2,3,5‑triphenyltetrazolium chloride (TTC) staining of zebrafish brain showed that the absorbance of the MgT-treated group was 1.70-fold higher than in the hypoxia group indicating a reduction of the infarct area. TTC staining of brain tissue highlights both the infarct area and mitochondrial activity, correlating with the severity of brain damage following a stroke insult. In brain mitochondria, TTC reacts with succinate dehydrogenase to produce red formazan [17]. Higher absorbance levels after TTC staining indicate a smaller infarct area and greater mitochondrial activity, both of which correspond to reduced brain damage.

Furthermore, Kim et al. [16] also demonstrated that MgT protects brain tissue after hypoxia injury by upregulating the excitatory amino acid transporter (EAAT4). Oxygen deprivation caused by IS leads to excessive glutamate accumulation in the synapse, resulting in excitotoxicity. As one of the glutamate transporters, EAAT4 regulates synaptic glutamate levels, thereby preventing its accumulation in the brain. Upregulation of EAAT4 could therefore reduce cellular excitability induced by hypoxia, offering neuroprotection against further brain damage [79]. Western blot analysis showed that pretreatment with MgT increased EAAT4 expression by 1.65-fold compared to the model group.

### Isoliquiritin

Isoliquiritin is a hydrophilic bioactive compound isolated from licorice (*Glycyrrhiza* glabra) root. Based on its structure, isoliquiritin is classified as a chalcone glycoside, derived from the flavonoid group [80]. Previous studies showed multi-pharmacological activities of this compound including anticancer, antidepressant, anti-inflammation, promoting wound healing, and enhancing angiogenesis, and anti-allergic [81–84].

Zhang et al. [36] exhibited the anti-IS action of isoliquiritin in the zebrafish model. In their recent work, isoliquiritine showed the ability to reverse behavioral impairment in a dose-dependent manner. Moreover, isoliquiritin protected the morphology of cells in the brain and served as a cerebral antioxidant agent by enhancing glutathione peroxidase (GSH-Px) activity (1.60–1.63-fold) and superoxide dismutase (SOD) activity (1.18–1.57-fold) and reducing malondialdehyde (MDA) activity (0.37–0.57-fold) after photothrombotic exposure. GSH-Px, SOD, and MDA are key oxidative stress markers in IS. Following IS, the blockage of blood flow in the brain leads to oxygen and nutrient deprivation, which increases oxidative stress and activates antioxidant enzymes such as GSH-Px and SOD. Inadequate antioxidant defense mechanisms result in elevated levels of ROS, leading to lipid peroxidation and increased MDA production, both of which contribute to neurodegeneration [85]. Thus, elevated levels of GSH-Px and SOD, alongside reduced MDA, indicate that isoliquiritin exerts neuroprotective effects by mitigating oxidative stress associated with IS.

In the study conducted by Zhang et al. [36], a transcriptomic analysis of telencephalon in the zebrafish IS model followed by Gene Ontology (GO) and Kyoto Encyclopedia of Genes and Genomes (KEGG) enrichment and real-time quantitative PCR (qRT-PCR) investigation was performed to explore isoliquiritin’s protective mechanism. The analysis identified 3,646 differentially expressed genes (DEGs), with the majority being upregulated in the model group compared to the sham group, and subsequently downregulated in the model + isoliquiritin group. GO and KEGG enrichment analyses indicated that isoliquiritin confers protection to membrane structure and function, and initiates immune regulation in response to stress post-ischemia. The protective mechanisms of isoliquiritin were particularly associated with immune-related pathways, apoptosis, and necrosis pathways. These findings were corroborated by qRT-PCR analysis, which demonstrated that the log2 fold change in gene expression patterns of 25 key DEGs observed with qRT-PCR was consistent with the RNA-seq results.

### Guhong injection

Guhong injection (GHIJ), a Traditional Chinese Medicine formulation, consists of extracts from safflower (*Carthamus tinctorius*) and N-acetyl-L-glutamine. Safflower is rich in diverse phytochemicals, including flavonoids, alkaloids, polyacetylenes, and organic acids, while N-acetyl-L-glutamine is a single compound. GHIJ has shown multiple therapeutic properties, such as anti-inflammatory, antioxidant, anticoagulant, antithrombotic, neuroprotective, and anti-apoptotic effects, making it beneficial for the treatment of IS [86].

Wang et al. [33] investigated the anti-stroke mechanism of GHIJ (2.5–7.5%) using zebrafish models of thrombosis and IS. In their study, erythrocyte fluorescence-labeled Tg (LCR:eGFP) zebrafish embryos were employed in a phenylhydrazine (PHZ)-induced thrombosis model to measure peripheral and cerebral blood flow. PHZ stimulation significantly reduced blood flow in the caudal vein and the brain's primary cephalic sinus, but this reduction was effectively counteracted by GHIJ. After GHIJ treatment, the erythrocyte flow rate increased by 4–11-fold compared to the PHZ group. Additionally, photoperiod treatment was used to assess the motor behavior of the experimental fish. Supplementation with GHIJ and its major compounds, baicalein, chlorogenic acid, gallic acid, and rutin, markedly improved the total distance traveled, average speed, and proportion of movement time of the zebrafish IS model by 2.00–8.40, 1.50–7.00, and 1.67–7.00-fold, respectively for each parameter. This result indicates that GHIJ exerts a protective effect on brain function.

Wang et al. [33] also reported that GHIJ and its bioactive components exhibit antithrombotic and anti-inflammatory properties in cerebral ischemia by regulating specific genes. In PHZ-induced thrombosis and ponatinib-induced IS models, there was a significant upregulation of coagulation factors. Supplementation of GHIJ and its active compounds downregulated the expression of those factors which were factor VII by 0.47–0.62-fold and factor II by 0.43–0.47-fold. However, the antithrombotic effects of GHIJ did not appear to involve the platelet activation pathway, as evidenced by the lack of effect on the expression of prostaglandin-endoperoxide synthase 1, prostaglandin-endoperoxide synthase 2a, and thromboxane A synthase 1. Conversely, GHIJ resulted in the downregulation of nuclear factor-κB, a crucial inflammatory regulator, in both the PHZ-induced thrombosis model by 0.85-fold and ponatinib-induced IS model by 0.59-fold, indicating its anti-inflammatory action through modulation of the inflammatory signaling pathway.

### Brazilin

Brazilin is the principal homoisoflavonoid compound derived from heartwood (*Caesalpinia sappan*) [87]. Heartwood has been traditionally employed in the treatment of IS. Meanwhile, as an isolated compound, brazilin exhibits a wide range of pharmacological activities, including antioxidant, antibacterial, anti-inflammatory, hepatoprotective, anti-hypoglycemic, vasorelaxant, anticancer, antidiabetic, anti-Alzheimer, anti-Parkinson, antidepressant, and anxiolytic properties [88–90]. Guo et al.[91] demonstrated that brazilin has an anti-stroke effect by enhancing locomotor activity in a zebrafish model. They observed a 1.50–1.70-fold improvement in total distance traveled in the group treated with brazilin. This neuroprotective effect was corroborated by in vitro studies using OGD/R-induced mouse primary neurons and neuro-2a cells, as well as in vivo experiments with a rat MCAO model. Brazilin treatment, at concentrations ranging from 1.5 to 6.0 µM, significantly reduced cell apoptosis due to OGD/R insult and improved cell viability in a concentration-dependent manner. Additionally, brazilin ameliorated neurological deficits, decreased brain infarction and edema, and protected against neuronal pathological changes induced by MCAO in rats. Both the zebrafish and rat models indicated that brazilin modulates deoxyhypusine hydroxylase activity, enhancing mitophagy in vivo.

### Minocycline

Minocycline, an antibiotic in the tetracycline class, has been investigated for potential repurposing in recent years. Studies have demonstrated that minocycline possesses neuroprotective properties beneficial for conditions such as IS, traumatic brain injury, subarachnoid hemorrhage, intracerebral hemorrhage, and depression [92, 93]. Notably, minocycline could penetrate BBB since it is lipophilic. As a clinically approved drug, minocycline is affordable and presents a minimal side effect profile that it has potential as a stroke therapy [94]. Research into the efficacy of minocycline for stroke treatment has been conducted using both rodent models and human clinical trials [95].

A previous study exhibited the neuroprotective effect of minocycline to counter ischemia and reperfusion injury in hypoxia-induced IS in the zebrafish model [35]. The TTC staining showed that the minocycline-treated group had recovery of the infarct area compared to the hypoxia group. This result indicates that minocycline improved mitochondrial metabolism in the brain after ischemia episodes. Furthermore, the swimming activity of experimental zebrafish was evaluated through several parameters including the distance traveled, absolute turning angle, and number of crossings. In this study, locomotor impairments occurred after hypoxia, and the administration of minocycline improved locomotor activity by 2.00, 1.64, and 2.40-fold for each parameter, respectively. In their study, de Medeiros Borges et al. [35] showed that the results obtained from the zebrafish IS model are consistent with in vitro and in vivo studies using rodent models.

### Qilong capsule

The Qilong capsule (QC) is a patented Chinese medicine derived from the "Buyang Huanwu decoction" with stringent quality control over its active ingredients. The China Food and Drug Administration approved the Qilong capsule for IS treatment in 2000, but its use is limited in China. The formulation of QC emphasizes both Qi replenishment and blood circulation activation, primarily targeting IS characterized by Qi deficiency and blood stasis syndrome. There are eight medicinal ingredients in QC such as *Astragalus membranaceus* (Huangqi), earthworm (Dilong), *Salvia miltiorrhizae* (Danshen), *Angelica sinensis* (Danggui), red peony root (Chishao), *Ligusticum chuanxiong* Hort (Chuanxiong), safflower (Honghua), and peach kernel (Taoren) [96]. Research has identified the principal active constituents of QC as astragalosides A, calycosin-7-glucoside, lumbrokinase, tanshinone IIA, salvianolic acid B, ferulic acid, paeoniflorin, hydroxysafflor yellow A, and amygdalin [97].

Lin et al. [50] demonstrated that QC (1.56–25 μg/mL) served as anti-IS in ponatinib-induced zebrafish model through various mechanisms including infarct area reduction, antiapoptosis, and antiinflammation, following a concentration-dependent manner. Treatment with QC markedly reduced the cerebral thrombosis area and red blood cell staining intensity by 46–69% and 50–71%, respectively. QC significantly decreased the number of apoptotic cells in larval brains treated with ponatinib by 0.29–0.68-fold and reduced the mRNA expression of apoptosis-related genes which were *caspase1*, *caspase2*, *caspase3*, *caspase7*, *caspase8*, *caspase9*, and *bax gene* by 0.41–0.73, 0.31–0.50, 0.38–0.50, 0.03–0.23, 0.08–0.17, 0.48–0.60, and 0.29–0.50-fold, respectively. Lin et al. also revealed that JS7 transgenic zebrafish, which express macrophages, exhibited macrophage accumulation in the brain tissue of the ponatinib group and the number declined by 29%-83% after QC treatment. In the QC-treated group, they also observed the down-regulation of inflammation-related gene expression which were *il-10*, *il-1β*, *p38*, and *nf-кb* by 0.20–0.68, 0.33–0.63, 0.24–0.59, and 0.30–0.61-fold, respectively. Furthermore, QC down-regulated the expression of coagulation-related genes which were *PCK-α*, *PCK-β*, *fga*, *fgb*, *f2*, and *vWF* by 0.22–0.39, 0.12–0.16, 0.08–0.15, 0.03–0.06, 0.39–0.78, 0.25–0.45, and 0.21–0.63-fold, respectively. This result marked the mechanism of the anti-stroke effect of QC is related to the inhibition of platelet activation and the coagulation cascade.

### *Caragana jubata*

*Caragana jubata* (CJ) is a plant that is highly valued in Tibetan, Chinese, and Russian traditional medicine [98, 99]. CJ exhibits multi-pharmacological activities such as hepatoprotector, antithrombotic, anti-inflammatory, antioxidant, and antimicrobial activities [100–103]. Flavonoids are the major component of this plant. The red heartwood part of CJ contains various sub-classes of flavonoids including isoflavones, flavones, flavonols, and flavanones [65]. A main compound of this plant, Texasin, showed a neuroprotective effect in the OGD/R-injured PC12 cell model [98].

Zhao et al. [65] conducted a study investigating the protective effects of the ethanolic extract of the red heartwood part of CJ in IS. Using a zebrafish model, they observed that CJ extract (2–50 μg/ml) reduced the incidence of cerebral thrombosis by 16–76% following ponatinib induction. Notably, the study highlighted that 50 μg/ml of CJ extract was more effective in reducing cerebral thrombosis incidence than 30 μg/ml of aspirin. The anti-IS effect of CJ ethanolic extract was also verified using OGD/R-induced PC12 or BV2 cell damage models and the MCAO/R rodent model.

### 6S-5-Methyltetrahydrofolate-Calcium

6S-5-Methyltetrahydrofolate-calcium (MTHF-Ca) is the crystalline form of the calcium salt of L-5-methyltetrahydrofolate (L-5-MTHF), which is the predominant form of dietary folate present in the bloodstream [104]. Upon ingestion, MTHF-Ca dissociates fully in an aqueous environment into Ca^2+^ and L-5-MTHF ions, which are then absorbed by the intestinal mucosa. L-5-MTHF serves as an alternative to folic acid in human food and dietary supplements. MTHF-Ca showed an anti-inflammatory effect on RAW264.7 cells and zebrafish [105]. Currently, L-5-MTHF and MTHF-Ca supplements are increasingly being investigated for their therapeutic potential in treating various conditions, including Alzheimer's disease, depression, and other mental and neurological disorders.

Bin et al. [17] investigated the anti-IS effects of MTHF-Ca in a photothrombotic-induced adult zebrafish model. Their findings indicated that MTHF-Ca (497.5 mg/kg) conferred protection against ischemic brain injury through multiple mechanisms. It reduced telencephalon injury score and rate by 24% and 20%, respectively. MTHF-Ca restored motor function through improvement of the total swimming distance, mean velocity, number of turn clockwise swimming, number of transitions to the top, and proportion of zebrafish activity in the tank by 2.67, 3.00, 3.48, 3.08, 5.38, and 2.17-fold, respectively. It played as an antioxidant agent via enhancement of GSH-Px activity by 1.12-fold and SOD activity by 1.15-fold, and reduction of MDA content by 0.55-fold. Moreover, transcriptomic analysis of the fish telencephalon, complemented by GO and KEGG enrichment analyses and RT-qPCR validation, revealed that the neuroprotective effects of MTHF-Ca are primarily mediated through the inhibition of neuroinflammation and the prevention of brain cell apoptosis, achieved by modulating the mitogen-activated protein kinase (MAPK) signaling pathway.

### Future perspective

The currently available zebrafish IS models are rudimentary, but many aspects can be improved to develop a model that is more adequate and relevant to clinical conditions. Tables 1 and 2 show that only a limited number of studies have focused on establishing a zebrafish IS model with comorbidities. Therefore, we suggest further establishment of more complex models, such as the zebrafish IS model with diabetes, as presented in a published study by Chavda et al. [28]. Considering that some models of zebrafish with hypertension, obesity, and hyperlipidemia are already available [106–110], efforts to improve these models are possible. Another important research gap that has yet to be resolved is pharmacokinetic and pharmacodynamic studies on interactions between comorbid drugs and prospective drugs for IS treatment.

On the other hand, the ongoing development of the zebrafish IS model is shaped by various factors that affect its consistency and reproducibility. Current methods for inducing ischemic stroke in zebrafish, such as mechanical occlusion or chemical approaches, often lack consistency. Moreover, differences in environmental and housing conditions across labs further impact reproducibility. Thus, standardizing the procedures for stroke induction and establishing uniform protocols for zebrafish care, housing, and experimental conditions including zebrafish strain and developmental period, are crucial. These steps will result in more accurate and reproducible models across different research settings.

Table 2 shows that zebrafish IS models have not been utilized to examine drug responses to sex and genetic discrepancy. Future exploration of this topic could contribute to personalized medicine for IS and ultimately enhance patient outcomes. Moreover, an experimental design using a zebrafish IS model that enables the observation of several important parameters for IS treatment such as cerebral angiogenesis and neurogenesis should be further explored. This development might adapt to the currently available procedures [111–116]. Investigating the neuro-regenerative mechanisms in zebrafish can also lead to innovative strategies for improving brain repair and recovery in stroke patients.

A primary goal of preclinical studies is to successfully translate findings from animal models to human clinical practice. Despite many genetic and physiological similarities between zebrafish and humans, significant differences in brain structure and function pose challenges for direct translation of findings. Thus, we recommend using integrated multi-model approaches. Discoveries made in zebrafish models should serve as initial stages of drug development and require validation in higher mammalian models before being applied to humans. Reaching this objective necessitates a more comprehensive understanding of zebrafish neuroanatomy and physiology to better match research findings with mammalian models. Although this adds an extra layer of research, it ensures that results are relevant and translatable to human physiology.

## Conclusions

The zebrafish IS model has been used in studying new therapeutic agents for IS such as magnesium L-threonate, isoliquiritin, Guhong injection, braziline, minocycline, Qilong capsule, extract of *Caragana jubata*, and 6S-5-methyltetrahydrofolate-calcium. Based on this review, we conclude that several key considerations must be addressed when using zebrafish as an experimental model for ischemic stroke (IS), including induction methods, developmental stages, zebrafish sex and strain, route of drug administration, and assessment parameters. While the zebrafish is a promising model for conducting preclinical studies on IS, we recommend that the results obtained from zebrafish studies be validated in mammalian models to ensure successful translational research.

## Data Availability

Not applicable.

## Abbreviations

AO: Acridine orange
BBB: Blood–brain barrier
BW: Body weight
DEGs: Differential expression genes
CAT: Catalase
CJ: *Caragana jubata*
DEGs: Differentially expressed genes
DO: Dissolved oxygen
DOI: Digital object identifier
dpf: Days post-fertilization
EAAT4: Excitatory amino acid transporter
ET-1: Preproendothelin-1
GHIJ: Guhong injection
GSH-Px: Glutathione peroxidase
GO: Gene Ontology
H&E: Hematoxylin–eosin
hpf: Hours post-fertilization
IL4R: Interleukin 4 receptor
IS: Ischemic stroke
i.p: Intraperitoneal
KEGG: Kyoto encyclopedia of genes and genomes
L-5-MTHF: L-5-methyltetrahydrofolate
MAPK: Mitogen-activated protein kinase
MCAO: Middle cerebral artery occlusion
MCAO/R: Middle cerebral artery occlusion/reperfusion
MDA: Malondialdelhyde
MgT: Magnesium L-threonate
MTHF-Ca: 6S-5-Methyltetrahydrofolate-Calcium
NA: Not available
OGD/R: Oxygen–glucose deprivation/reoxygenation
PHZ: Phenylhydrazine
qRT-PCR: Real-time quantitative PCR
ROS: Reactive oxygen species
r-tPA: Recombinant tissue plasminogen activator
p.o: Per oral
ROS: Reactive oxygen species
SOD: Superoxide dismutase
STAIR: Stroke therapy academic industry roundtable
TTC: 2,3,5‑Triphenyltetrazolium chloride
QC: Qilong capsule
